# Comment on “The Role of Citrullinated Protein Antibodies in Predicting Erosive Disease in Rheumatoid Arthritis: A Systematic Literature Review and Meta-Analysis”

**DOI:** 10.1155/2015/175251

**Published:** 2015-08-23

**Authors:** Shailendra Kapoor

**Affiliations:** Private Practice, 67 Crossing, Richmond, VA 23345, USA

The past few years have seen the identification of new biomarkers that have shown considerable promise [1] in assessing disease severity in patients with rheumatoid arthritis (RA).

Plasma neopterin is an emerging and promising marker to assess disease activity in patients with RA [2, 3]. For instance, early RA patients have a mean neopterin concentration of 8.92 nmol/liter in comparison to a corresponding mean level of 5.63 nmol/liter in non-RA individuals. This has been confirmed recently by Arshadi et al. who have reported accentuated neopterin levels in patients with the active stage of RA [4]. In addition, men with RA tend to exhibit high neopterin levels when compared to neopterin levels noted in women [5]. D'agostino et al. have reaffirmed this relationship by confirming the direct relationship between DAS-28 scores and serum neopterin levels [6]. Interestingly, RA patients who are negative for anti-CCP antibodies tend to have lower neopterin levels when compared to patients who are positive for anti-CCP antibodies [7]. Age at the time of initial presentation of RA also has a significant impact on neopterin expression. Macrophage derived soluble CD163 (sCD163) is another newly identified marker of disease activity in RA. For instance, patients with RA have higher sCD163 levels when compared to sCD163 levels in patients with joint diseases such as osteoarthritis [8]. Greisen et al. in a recent study have demonstrated a significant decline in sCD163 concentrations following the initiation of appropriate RA therapy. In their study, the median sCD163 level decreased to 1.28 milligrams/liter when compared to an initial median sCD163 level of 1.69 milligrams/liter noted in patients with early stage RA [9]. In fact, a close association exists between the progression of RA related radiological anomalies and sCD163 levels. Interestingly, a close association also exists between serum CRP levels and sCD163 levels [10].

Assessment of serum YKL40 levels is also emerging as a sensitive marker to evaluate disease severity in RA, especially in the early stages [11, 12]. For instance, patients with early RA have higher YKL40 levels when compared to non-RA individuals [13]. A close association exists between the YKL40 levels and the number of joints involved in active RA. Interestingly, TGF-beta seems to have a negative impact on serum YKL40 levels [14]. Another new marker that has shown considerable promise is serum CXCL13 [15]. This has been confirmed by Meeuwisse et al. who have demonstrated accentuated rates of RA associated bone destruction in RA patients with higher CXCL13 levels [16]. Interestingly, this relationship is especially more significant in anti-CCP-2 negative patients.

The above markers have shown considerable promise so far. Hopefully, the coming few years will see their increased use in day to day rheumatology.
